# First Recorded Loss of an Emperor Penguin Colony in the Recent Period of Antarctic Regional Warming: Implications for Other Colonies

**DOI:** 10.1371/journal.pone.0014738

**Published:** 2011-02-28

**Authors:** Philip N. Trathan, Peter T. Fretwell, Bernard Stonehouse

**Affiliations:** 1 British Antarctic Survey, Cambridge, United Kingdom; 2 Scott Polar Research Institute, University of Cambridge, Cambridge, United Kingdom; University of Plymouth, United Kingdom

## Abstract

In 1948, a small colony of emperor penguins *Aptenodytes forsteri* was discovered breeding on Emperor Island (67° 51′ 52″ S, 68° 42′ 20″ W), in the Dion Islands, close to the West Antarctic Peninsula (Stonehouse 1952). When discovered, the colony comprised approximately 150 breeding pairs; these numbers were maintained until 1970, after which time the colony showed a continuous decline. By 1999 there were fewer than 20 pairs, and in 2009 high-resolution aerial photography revealed no remaining trace of the colony. Here we relate the decline and loss of the Emperor Island colony to a well-documented rise in local mean annual air temperature and coincident decline in seasonal sea ice duration. The loss of this colony provides empirical support for recent studies (Barbraud & Weimerskirch 2001; Jenouvrier et al 2005, 2009; Ainley et al 2010; Barber-Meyer et al 2005) that have highlighted the vulnerability of emperor penguins to changes in sea ice duration and distribution. These studies suggest that continued climate change is likely to impact upon future breeding success and colony viability for this species. Furthermore, a recent circumpolar study by Fretwell & Trathan (2009) highlighted those Antarctic coastal regions where colonies appear most vulnerable to such changes. Here we examine which other colonies might be at risk, discussing various ecological factors, some previously unexplored, that may also contribute to future declines. The implications of this are important for future modelling work and for understanding which colonies actually are most vulnerable.

## Introduction

Emperor penguins breed in coastal locations around the Antarctic with most colonies assembling on stable fixed or ‘fast’ ice, with just three occurring on land. All colonies show a similar breeding schedule regardless of colony location. Birds gather at traditional sites in autumn, with the development of the stable fast ice, usually from April onwards. Courtship, egg laying and incubation take place as winter proceeds, while hatching, brooding and crèche formation occur as winter abates and spring and early summer approach. Chicks are tended by both parents until fledging occurs in mid-summer, usually during November or December coincident with the breakup of stable fast ice into ‘pack’ (i.e. ice floes that drift with the winds and currents); however, chicks may still also be fed while taking refuge on drifting ice floes. Adults moult in late summer, during February, again usually on fast ice or on consolidated pack. Thus, emperor penguins depend upon stable fast ice for approximately eight months of the year, so late fast ice formation in winter and/or early breakup in spring can strongly reduce the chances of successful breeding at any given colony location.

Recently, changes in sea ice duration and distribution, associated with climate change, have been reported as important factors affecting emperor penguin population processes [1]-[6], with the main drivers of change thought to be reductions in sea ice [6]. Sea ice generally refers collectively to both fast ice and pack ice. Fast ice forms around the fronts of ice shelves, coastlines and between island archipelagos and grounded icebergs. It also forms over shoals or over shallows, and unlike pack ice, fast ice does not move with surface currents or wind [7]. At almost all locations emperor penguins use fast ice as a stable platform on which to breed [6], [8], [9] and since they are not agile, they are restricted to fast ice with a low free-board [4] that does not stand far above the ocean surface by more than a few tens of centimetres. Emperors may forage in polynyas, tide cracks and leads and within the pack ice so other consequences of climate change might also be important, particularly changing relationships with the sea ice community, including with their prey [4] which in most locations are fish, principally Antarctic silverfish, krill and squid [10].

Notwithstanding, conclusions about emperor penguin biology and ecology in relation to environmental change are limited by a severe lack of long-term population monitoring data and associated environmental data including, *inter alia*, data on prey species, predation and local and regionally scaled sea ice indices. Detailed demographic studies are currently only available from just one site [1]-[3] and population counts from only a few others. Consequently, without coherent monitoring programmes that examine demographic parameters in the context of a suite of environmental indices, inferences are necessarily limited and some conclusions may be merely correlative. Until more widespread demographic monitoring is available, previously unreported long-term time series of population counts from additional breeding sites might help increase our understanding of important ecological interactions between population processes and environmental drivers, particularly where population trajectories can be examined in the context of local environmental change.

One location where emperor penguin breeding population counts have been made but never previously published is at Emperor Island in the Dion Island group, Marguerite Bay, west of the Antarctic Peninsula. This site is one of the more northerly emperor penguin breeding locations; it was first discovered in 1948 [11], after which breeding counts were carried out on a sporadic and opportunistic basis, showing that the breeding population was relatively small, but stable. The demise of the colony from 1970 onwards was extremely rapid taking just over 30 years, comparable to the average lifespan of an individual [12] (∼20 years) but less than the life span of the longest free-living emperor (∼40 years). Here we argue that the most likely cause of this decline is a chronic reduction in the duration of sea ice from about 1970 onwards, associated with local atmospheric warming [6].

Though studies [1]-[6] have highlighted the importance of warming to the success of emperor colonies, mechanisms for exactly how such warming affects individual colonies remain unclear. In the case of the Emperor Island colony, the duration of seasonal pack ice has decreased at a rate of approximately four days per year at the colony site, one of the highest rates of loss recorded in the Antarctic in recent decades [13]. Implications are that atmospheric warming has reduced annual pack ice duration [14] and potentially stable fast ice, leaving the Emperor Island colony with reduced breeding habitat and vulnerable to ocean swell during late winter and spring storms.

At Emperor Island, emperors used to breed on the low rock and shingle isthmus at the southeast end of the island, one of only three colonies known to breed on land. This colony therefore also offers opportunities to speculate about how a range of other environmental drivers might also have affected the population decline.

## Materials and Methods

In this study, we combine breeding count data from an irregularly visited emperor penguin colony with locally recorded air temperature data, locally observed fast ice duration data and remotely sensed circumpolar sea ice duration data to evaluate the impacts of changing environmental conditions on breeding population size. Information on breeding counts, local air-temperature and local fast ice conditions were obtained from unpublished Falkland Island Dependency Survey (FIDS) and British Antarctic Survey (BAS) reports archived at BAS. Circumpolar sea ice duration data were obtained from the literature [13].

**Figure 1:**
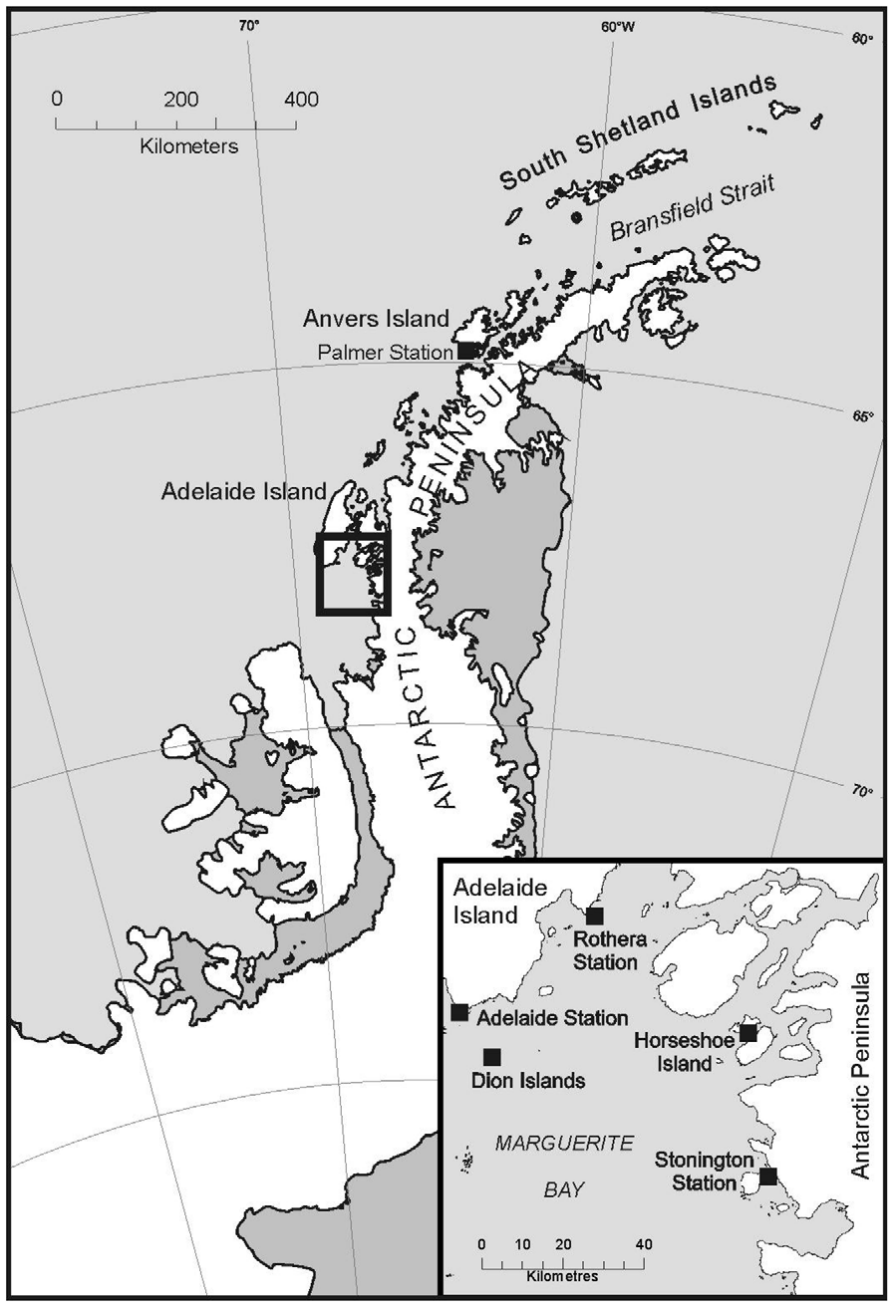
Map of the Antarctic Peninsula showing the study area inset.

Our study colony (67° 51′ 52″ S, 68° 42′ 20″ W) was located on Emperor Island in the Dion Island group, in Marguerite Bay, west of the Antarctic Peninsula (Figure 1). Emperor Island is just under 400 m by 300 m in dimension; at the north-western end it raises to 46 m whilst the eastern end is generally below 5 m. Emperors used to habitually breed on a low rock and shingle isthmus at the southeast end of the island. This colony is of historic interest as it was the fourth emperor colony to be discovered [11] and the location where the first study of breeding behaviour and early chick growth was carried out [15].

### Emperor penguin population counts

The numbers of adults, eggs and chicks at Emperor Island (Table 1) have been counted at irregular intervals since the colony was first discovered. Early in the time series, counts were taken in the middle of the winter when the fast ice could bear the weight of a man or sledge. Later in the time series other methods of access were used, including access by yacht or ship. Counts of the different demographic categories were recorded or estimated and are documented in a variety of FIDS/BAS base reports and published accounts; here we compile all the known counts for the first time. The source for each count is provided in Table 1. Where a population range was recorded we have taken the upper value as illustrative; taking the lower value, or an average, would result in the same conclusions. Counts for different demographic categories depended upon the timing of each visit. At Emperor Island, emperor penguins lay their eggs within a short time window in early June, and as only a single egg is laid, the best estimate of the breeding population in any given year would be of incubating birds just after laying was complete. However, a visit at this time of year was not always possible, due to both fast ice conditions and other logistic and operational constraints. Egg and offspring mortality occur cumulatively throughout the breeding season, therefore in any given year, counts in the middle of winter will be more representative of the breeding population than counts later in the season. Counts of incubating and brooding adults and of free-standing chicks made from July onward probably underestimate the size of the colony, but at least provide valuable minimum figures. Counts in October and November during the crèche stage may be less representative. However, given the clear trajectory of the population after the 1970s, and the difficulty in accessing Emperor Island after BAS closed a number of nearby bases, we consider that these late-season counts provide additional valuable information about the demise of the colony.

**Table 1:** Estimated number of emperor penguin breeding pairs on Emperor Island.

Date of visit	Number adults	Number chicks	Comment	Source reference
1948, October	NA	70		[15]
1949, 5 June–15 August	100–183	c. 150	Colony partially on sea ice	[15]
1957, 9 October	90	30 – 40	Colony on sea ice	[16]
1958, 27 July	160–170	Incubating		[17]
1963,13 August	150–200	Mostly incubating		[18]
1964, 9 July	200	Incubating		[Pers. comm. B Pimm-Smith]
1966, August	250	125		[19]
1968, 30 August	250	68		[20]
1969, 25 July	200	Incubating	Colony partially on sea ice	[21]
1970,12 August	250	Chicks on feet		[22]
1978, End of July	70–90	Incubating		[23]
1978, 3 November	85	20	Colony on sea ice	[23]
1999, 1 July	16	15 incubating		[Pers. comm. WR Fraser]
2001, 3 August	10	9 incubating		[Pers. comm. WR Fraser]
2009, 28 November	0	0		BAS aerial photography

Shaded cells indicate counts made later in the season, potentially after some egg/chick loss.

### Air temperature

No surface air temperature records exist for the Dion Islands (apart from records collected during the first study of breeding and early chick growth [15]), but a reliable temperature record exists from the FIDS/BAS base on Adelaide Island (67° 46′ S, 68° 55′ W; occupied 3 February 1961 to 1 March 1977) for the period from May 1962 until December 1974 (see www.antarctica.ac.uk/met/READER/surface/Adelaide.All.temperature.html; accessed on 7 October 2010). This is the geographically closest (∼12 km) air temperature record to the Dion Islands for the 1960s and 1970s.

After the base at Adelaide Island was closed, a BAS base at Rothera Point (67° 34′ S, 68° 08′ W; occupied 1 February 1976 until the present date) was opened and this now provides the geographically closest (∼40 km) air temperature record for the most recent decades (see www.antarctica.ac.uk/met/READER/surface/Rothera.All.temperature.html; accessed on 7 October 2010). The continuous temperature record has missing values for July and August 1999, but is analysed here from April 1977 until April 2010.

### Fast ice

No *in situ* sea ice records exist for the Dion Islands, but the islands are visible in good weather from the old base on Adelaide Island. Sea ice observations were made from Adelaide Island whilst it was occupied. Sea ice conditions around the Dion Islands were recorded from 1961 until 1973. The quality of the annual record was variable between years and sea ice conditions were not always recorded at regular and frequent intervals. Thus, it is not possible to always determine when periods of fast ice occurred and when these blew out as a result of winter storm activity. However, the date of the first fast ice formation was always recorded. Similarly, the date of the last fast ice formation was also recorded, although we assume that the last fast ice also existed until the day before the next chronological record when the fast ice finally blew out or disintegrated into shifting pack ice.

## Results

Though the breeding count data for emperor penguins at Emperor Island are irregular and often infrequent they provide an extremely valuable additional dataset for assessing emperor penguin population trajectories. The data are certainly adequate for assessing the size of the colony until 1970, and for indicating the approximate timing and rate of subsequent decline. Similarly, the data on local air temperature and local fast ice duration are intermittent and in particular do not cover the full period during which the breeding count data began to show a decline, nevertheless and in spite of these deficiencies, these data are unique and sufficient to support ecological associations in an exploratory and subjective manner.

### Emperor penguin population counts

The emperor penguin colony at Emperor Island was only visited in years when fast ice was sufficient to support a transit across, it was therefore only visited in some years, and then generally only during July or August, close to the coldest part of winter. No visits were generally feasible from September onwards during either chick rearing or fledging which generally takes place during December.

**Figure 2:**
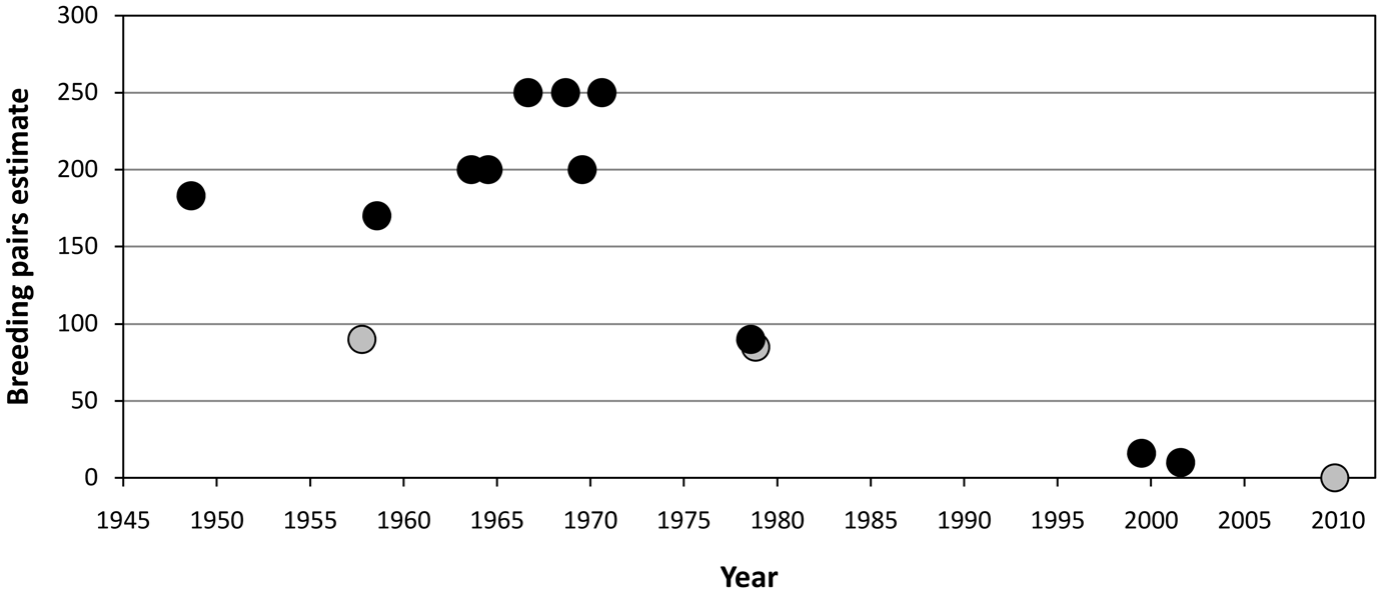
Estimated number of emperor penguin breeding pairs on Emperor Island. Black circles indicate counts made during winter and grey circles counts made later in the breeding season during spring, potentially after some egg/chick loss.

Counts of the numbers of adults, eggs and chicks at Emperor Island (Table 1) were undertaken sporadically, but occurred at least twice a decade after it was discovered until the end of the 1970s. Though colonies are known to vary inter-annually [1]-[3], the population at Emperor Island was relatively stable during this period (Figure 2).

After the base on Adelaide Island closed, counts were carried out less frequently, though it is during this period that the population started to show a monotonic decline (Figure 2). The decline appears to have started in the early 1970s, between 1971 and 1978.

### Air temperature

**Figure 3:**
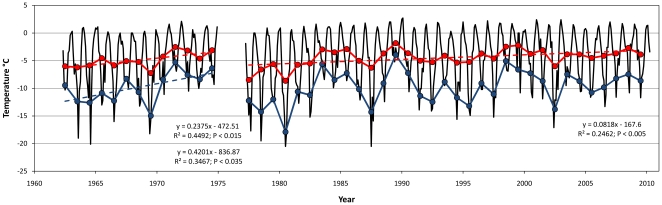
Variability in air temperature at Adelaide Island (May 1962 to December 1974) and at Rothera Point (April 1977 to April 2010). Thin black lines show the average monthly data; data from www.antarctica.ac.uk/met; red lines are the average annual (arithmetic average of all months) data; and blue lines average winter (arithmetic average of JJA) data. Analyses shows a significant trend in the average annual (dashed red line; F = 8.97; P<0.015) and average winter temperatures (dashed blue line; F = 5.84; P<0.035) at Adelaide Island and in the average annual temperatures (dashed red line; F = 10.13; P<0.005) at Rothera Point.

In our air temperature analysis (Figure 3) we show the average monthly mean (where the percentage of raw observations are adequate to calculate an accurate average), the average annual mean (the arithmetic average of all monthly average values), and the average winter mean (the arithmetic average of June, July and August) air temperatures. Simple linear regression analyses show a significant positive trend for both the average annual and average winter temperatures at Adelaide Island and for the average annual temperatures at Rothera Point. The increases in temperatures occur from the start of each time series, consistent with the known warming trend over the last century [24], [25].

### Fast ice

**Figure 4:**
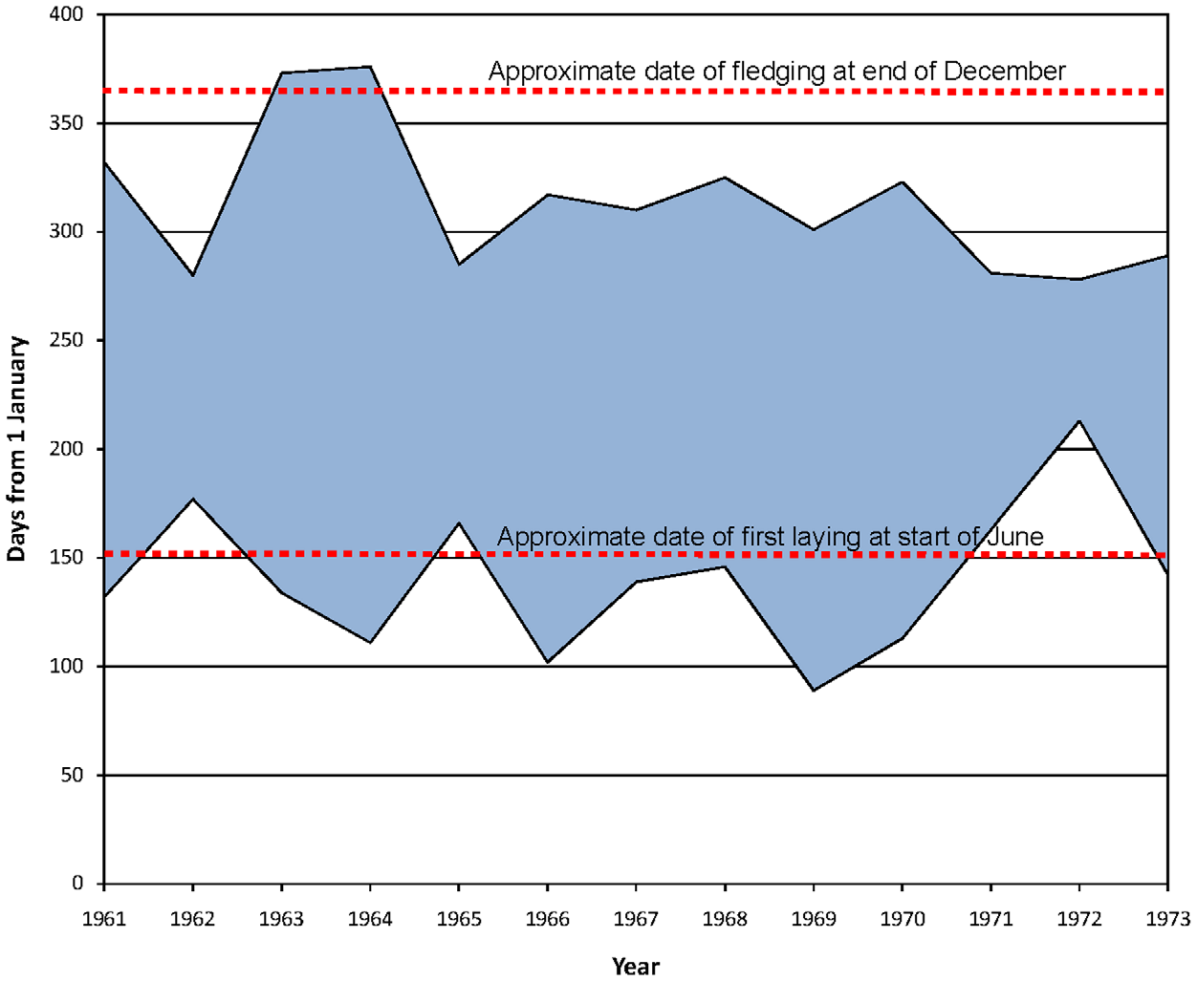
Inter-annual changes in the date of fast ice formation and break-up at the Dion Islands, observed from Adelaide Island, West Antarctic Peninsula; data from reports in BAS archives. The estimated dates for first egg-laying and fledging are shown.

Archived reports from the old station on Adelaide Island record the dates when fast ice formed and dispersed around the Dion Islands (Figure 4). The mean date of first fast ice formation in the area during the 1960s and early 1970s was 21 May (S.D 33.7 days), some days before the onset of egg laying and incubation in early June. The mean date of breakout was 10 November (S.D 33.0 days), at least a month before chick fledging in late December. The duration of the fast ice recorded for the Dion Island group (Figure 4) shows that the winter sea ice conditions were generally marginal for successful emperor penguin breeding and that the emperor penguin colony on Emperor Island relied upon the availability of land in most winters, even when the population size was relatively stable up until the 1970s.

### Circumpolar emperor penguin site variability

**Figure 5:**
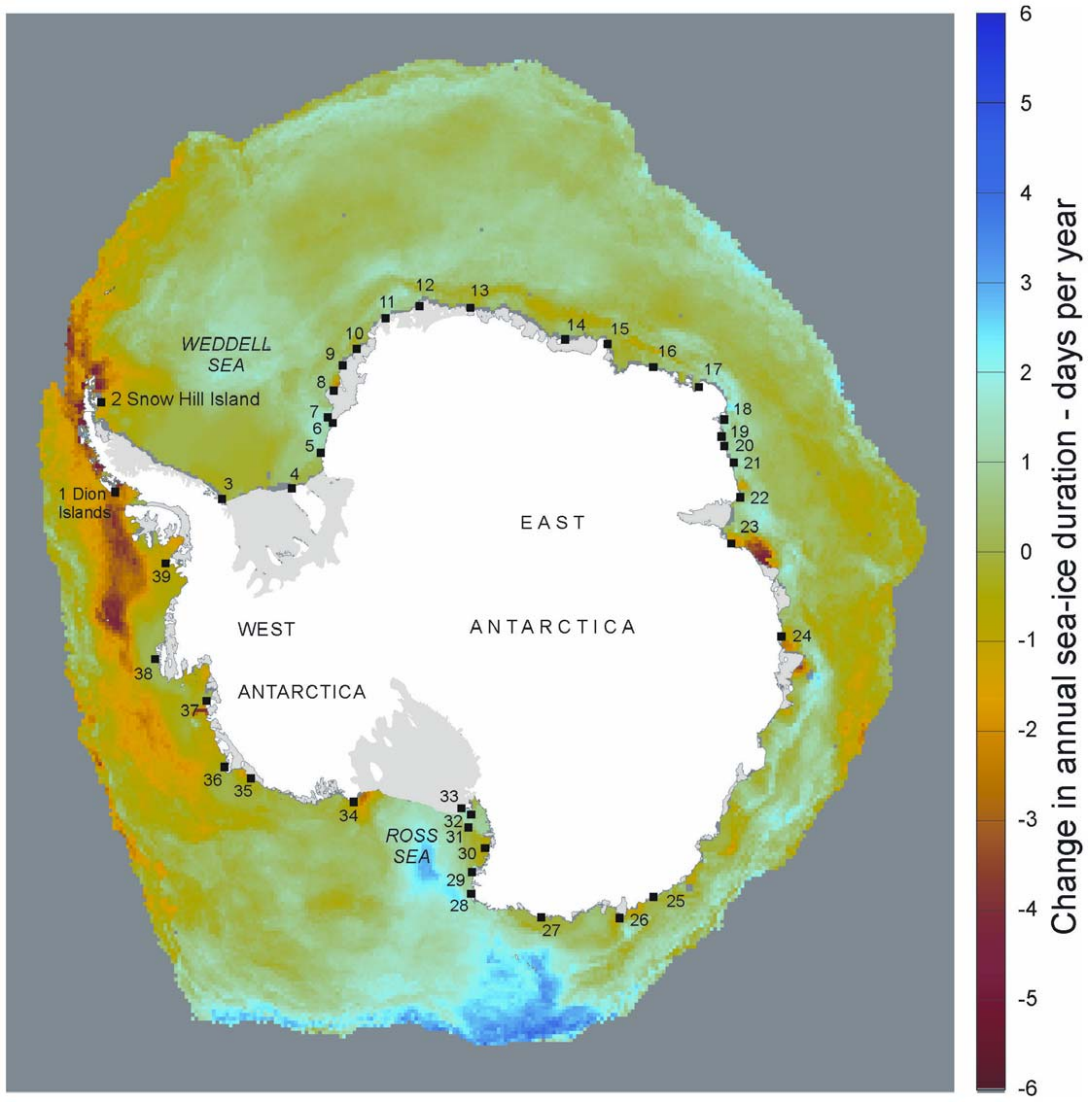
The spatial pattern of trend in sea ice duration change over 1979–2004 [13]. Scale shows trend in days per year. Black squares indicate the location of all known emperor penguin colonies [8]. Colony reference numbers refer to Table 2.

The link between fast ice and pack ice is complex and detailed relationships depend upon local circumstances; however, in most situations fast ice only forms in stable conditions, with surface currents, ocean swell and wind action potentially impeding fast ice development. Therefore, changes in fast ice production would be expected to occur if altered pack ice extent and duration meant locations were subject to increased wave action. No large-scale historical datasets exist for fast ice extent or duration, though remotely sensed data do exist for seasonal sea ice. The emperor penguin breeding locations (Figure 5) of all known colonies [8] can be overlaid onto recently published sea ice duration maps [13] to examine their vulnerability. Tabulation (Table 2) of sea ice duration [13] indicates those colonies that may be currently more vulnerable to climate change effects. The colony at Emperor Island is where sea ice trends are most negative. However, the colony is not the most northerly emperor penguin breeding site, there being 9 others that occur at lower latitudes. Similarly, colony locations where sea ice trends are most positive are not the most southerly, there being a number that are further south; it is notable that those colonies where sea ice trend is most positive all occur in the Ross Sea.

**Table 2:** Emperor penguin colony locations in relation to trends in sea ice duration.

	NAME	LONGITUDE	LATITUDE	SEA-ICE TREND AT COLONY
		(decimal degrees)	(decimal degrees)	(days per annum)
1	Dion islands	−68.70	−67.87	−4
2	Snow Hill Island	−57.46	−64.52	0
3	Smith Peninsula	−60.85	−74.38	0
4	Gould Bay	−47.32	−77.74	0
5	Luitpold Coast	−33.65	−77.27	0
6	Dawson Lambton	−26.56	−76.01	0
7	Halley Bay	−27.20	−75.52	0
8	Stancomb Wills	−23.02	−74.16	0
9	Drescher Inlet	−19.12	−72.86	0
10	Kitkuven (RI ice shelf)	−15.13	−72.14	0
11	Atka Bay	−8.13	−70.62	0
12	Sanae	−1.38	−70.05	0
13	Astrid Coast	8.31	−69.94	0
14	Ragnhild Coast	27.25	−69.97	0
15	Riiser Larsen Pen(Gunn)	34.39	−68.78	0
16	Umbeashi Rock	43.12	−68.05	0
17	Amundsen Bay	50.74	−66.77	0
18	Kloa Point	57.30	−66.64	0
19	Fold Glacier	59.38	−67.33	0
20	Taylor Glacier	60.88	−67.48	0
21	Auster	64.00	−67.40	0
22	Cape Darnley	69.70	−67.88	0
23	Amanda Bay	76.88	−69.28	−1
24	Haswell Island	93.01	−66.53	−1
25	Point Geologie	140.01	−66.67	0
26	Mertz Glacier	146.45	−66.93	0
27	Davies Bay	158.41	−69.33	0
28	Cape Roget	170.56	−71.98	1
29	Coulman Island	169.64	−73.34	1
30	Cape Washington	165.38	−74.65	0
31	Beaufort Island	167.04	−76.94	0
32	Franklin Island	168.40	−76.18	1
33	Cape Crozier	169.43	−77.51	1
34	Edward VII Pen	−157.74	−77.13	0
35	Ledda Bay	−131.57	−74.36	0
36	Thurston Glacier	−125.59	−73.43	0
37	Bear Peninsula	−110.17	−74.37	0
38	Noville Peninsula	−98.49	−71.75	0
39	Smyley Island	−78.75	−72.31	0

Locations for all known colonies are taken from published analyses [8]. Sea ice trend values are also taken from recent analyses [13], with values for each colony location represented by the geographically closest pixels. Shaded values indicate colonies that are in areas of current significant sea ice loss.

## Discussion

### Demise of the Emperor Island colony

Emperor penguins breeding in the West Antarctic Peninsula region may be more vulnerable to climate change that colonies elsewhere on the continent. For much of the twentieth century the climate of the West Antarctic Peninsula region has warmed at an unprecedented rate [24], [25]. This has been particularly rapid in recent decades [26], [27]. The recent warming has been ascribed to changes in atmospheric circulation over the Southern Ocean [14], [28]. The westerly winds that surround Antarctica have increased by around 15–20% since the 1970s [14], [28]. These stronger winds bring northerly oceanic warm wet air into the Amundsen-Bellingshausen Sea region. These conditions have now resulted in a clear dipole in seasonal sea ice with significant positive trends in the Ross Sea and significant negative trends in the Amundsen-Bellingshausen Sea [14]. Regional warming caused by intensification of the westerly winds has reduced seasonal sea ice and even led to ice shelf collapse along the eastern edge of the Antarctic Peninsula [28], [29]. Overall, 87% of the Peninsula's glaciers have retreated in recent decades [30]. At the same time surface waters are also known to have warmed [27]. Air temperatures at Rothera station, 40 km to the north of Emperor Island, have shown a very substantial warming trend [31], although the duration of the temperature record and large inter-annual variability in temperatures driven in part by the El Niño-Southern Oscillation means that this trend is not always statistically significant.

At the Dion Islands, FIDS/BAS archived reports show that in some years, fast ice only formed after emperor penguins began to lay their eggs, while fast ice usually blew out or melted before any chicks fledged. For some years the archived reports also included details of ocean swell, indicating when sea ice was limited and insufficient to dampen wave action in the area. The shingle isthmus on which the colony existed was formed by storm action and as such would be vulnerable to the significant increase in severe storm events reported for the Peninsula region [32], particularly if unprotected by sea ice during winter. Even up until 1970, the Emperor Island colony must have been a site of high risk for both incubating birds and chicks, perhaps accounting for the relatively small size of the colony. Thereafter annual hazards must have increased as pack ice became progressively less extensive and of shorter duration offering less damping to wave action.

During several visits to Emperor Island, the colony was recorded wholly or partially (June 1949, July 1969, October 1957 and November 1978) on fast ice (Table 1) and it may have existed on fast ice in other years. When restricted to land, as opposed to the surrounding fast ice, breeding habitat would be more limited, potentially reducing an individual's ability to escape the worse extremes of the wind and to access fresh snow to reduce any increasing effects of dehydration [33]. This could have physiological implications for males during incubation and for chicks after hatching and prior to fledging. Such restrictions may then limit chick survival and colony size, as has been suggested previously for other small colonies at Cape Crozier, Beaufort Island and Franklin Island [33].

Warming and reduced sea ice duration may also have led to changes in a number of important trophic interactions that might have contributed to the decline of the colony. Changes in fish, krill and squid communities on which emperor penguins feed are likely to be important drivers of population change [4], especially when prey stocks are reduced locally by loss of sea ice [34], and particularly within their foraging ambit when emperors act as central-place foragers. An additional factor that has not been noted previously is the possibility of increased predation. Emperor penguins breed so far south that chicks are generally well advanced before raptorial seabirds commence breeding. However, in October 1957 giant petrels (*Macronectes giganteus*) were predatory on the group; their nearest known breeding site is at Avian Island, 12 km away. With warmer, earlier springs we speculate that giant petrels or other raptorial seabirds could be more active at some of the more northerly emperor colonies. Future monitoring at emperor colonies should therefore also include monitoring of nearby raptorial seabird species.

**Figure 6:**
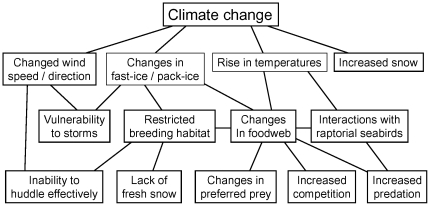
Partial ecogram summarising climate change effects: potential ecological drivers of change in emperor penguin populations.

The number of ecological processes affected by climate change is extensive, including for processes operating at different times of year and across all life history stages. In Figure 6 we show a partial ecogram summarising some of the important potential ecological drivers of change.

The rate of climate change in the West Antarctic Peninsula region has been extremely rapid [35], almost certainly beyond levels of phenotypic plasticity exhibited by penguins over millennial timescales [36], [37]. Indeed, it has been suggested [38] that the most likely response of penguins to climate change is dispersal, recognising that adaptation and in particular microevolution in penguins is a slow process, or at least, punctuated. Thus, emperor penguin population processes at Emperor Island might be expected to exhibit this same pattern; that is, that population numbers declined in response to climate change as recruitment to the colony became increasingly uncommon. Breeding habitat availability (on land) will have changed little over the past 50 years, and emperor penguins with their long lifespan might have remained site-faithful long after environmental conditions became sub-optimal. Thus, we speculate that recruits from the late 1970s may have continued to breed, in ever decreasing numbers, up until the start of this century. There is little information available about how emperors disperse and recruit, but given the vulnerability of their fast ice breeding habitat to storm action, breeding pairs may move between sites and juveniles may recruit to other sites rather than to their natal colony. Each colony is thought to form within a few kilometres of its traditional location [39]; however, the instability of ice shelves and ice tongues means that emperor colonies located close to such features must periodically relocate, even if only a short distance. For example, the recent break-off of the Mertz Glacier Tongue in February 2010, means that the emperor colony located close to this features [8] must now relocate. All existing demographic studies suggest that emperors are philopatric [1]-[3], but with changing, though not necessarily catastrophic, conditions, recruitment of juveniles might also be to other sites. Monitoring of juvenile recruitment with respect to their choice of breeding site must now be a priority for future research.

### Generic considerations

The ability of emperors to accommodate changes in patterns of climate and environmental variation is likely to be critically important [3], [40]. However, understanding the altered ecological interactions driving a population's vital rates is also key. When breeding on land emperors would not have an obligate requirement for the stable substrate of fast ice, therefore it is plausible that other consequences of climate change, such as the reduced duration and extent of pack ice, might mean that altered food-web connections may be more critically important. Although the other land-based colonies, Taylor Glacier (67° 40′ S, 60° 53′ E) and Amundsen Bay (66° 46′ S, 50° 45′ E) [8], do not appear to be vulnerable to current changes in sea ice, the demise of the Emperor Island colony shows that shifting their breeding habitat to accessible low-lying land to mitigate against the loss of sea ice might not be a long term survival option for this species.

Recent studies indicate that reductions in sea ice associated with climate change will have profound consequences for emperor penguin populations [3], [4]. Currently such studies do not expressly discuss projections of fast ice and rely upon projections of sea ice. Given the complex local association between protective pack ice [7] and fast ice, changes in pack ice extent and duration will potentially not only alter the character of stable breeding habitat, but also and importantly, the foodweb connections within the pack itself. Results from Emperor Island suggest that this may be vitally important.

Current assessments suggest that the observed di-pole in sea ice between the Antarctic Peninsula and the Ross Sea will alter as the ozone hole heals in the second half of this century [14], [28]. Contributory factors continue to be debated [41] and refined, but current predictions [14], [28] suggest that trends in sea ice will alter and the annual average sea ice extent will diminish by 33%; most of this retreat will be in winter and spring [28], with attendant risks for emperors.

Of fundamental importance to future forecasts of emperor penguin colony viability will be an increased ecological understanding of how they utilise their available habitat and therefore how environmental changes will affect both their use of habitat and their behaviour. For example, the wider regional pack ice, in terms of the level of convergence/divergence, seasonality and concentration, will impact on the well-being of non-breeding birds and breeding birds both during and outside the breeding season. Changes in pack ice extent and duration may affect predictable foraging grounds that are within foraging range of each breeding or moult location; similarly changes in polynya extent and persistence will almost certainly be an important factor. Also, knowledge about fast ice production is a potentially crucial factor for breeding and possibly moult. At present there are fundamental gaps in our knowledge about the types of sea ice features that emperor penguins rely upon. Similarly, current climate models do not represent most of these sea ice features to the extent required for future habitat modelling. Coupled atmosphere-ocean models are the foundation for most climate initiatives. However, the models have many problems in simulating sea ice extent, as small errors in the atmospheric circulation or oceanic conditions can give large errors in the sea ice extent and area [42]; fast ice and polynyas are even more difficult to model.

### Alternative hypotheses

In this paper we have considered the demise of the emperor penguin colony on Emperor Island in relation to climate change. However, alternative plausible explanations might be used to interpret the reduction in numbers at the colony. Such alternatives are essential to consider if we are to avoid incorrectly diagnosing threats to potentially vulnerable species, or indeed individual colonies, and if we are to understand the influence of regional change on marine ecosystems [43], [44]. Here we consider five alternative, but not mutually exclusive hypotheses that might have led to the demise of the colony on Emperor Island.

Firstly, increased snowfall consequent on increased warm, wet conditions may have contributed to population declines [9]. Such a situation has been reported for the West Antarctic Peninsula close to Palmer Station, Anvers Island (64° 46′ S, 64° 03′ W) [35] where increased snow precipitation has affected Adélie penguin colonies breeding on landscapes where snow accumulations are enhanced by landscape aspect and prevailing winds during spring storms; these colonies have decreased significantly faster than colonies where wind scour abates snow accumulations. However, no snowfall records exist for Emperor Island and no previous impacts of increased snowfall have been noted for emperors.

Secondly, short, but extreme weather events could also have led to the demise of the colony. Such extreme events have been reported for the West Antarctic Peninsula region [45]; they occur periodically and have impacts across a wide range of trophic levels, including for penguin populations. Further, but related to this are anomalous periods of weather that last over longer timescales. For example, between 1975 and 1980 there was a step change in the population size of the emperor penguin colony at Pointe Géologie, Dumont d'Urville, Terre Adélie (66° 40′ S, 140° 01′ E) during a prolonged abnormally warm period with reduced sea ice extent [1]. Emperor mortality rates increased when warm sea-surface temperatures occurred in the foraging area and when annual sea ice extent was reduced, and were higher for males than for females. Interestingly, the start of the colony decrease at Emperor Island began between 1971 and 1978 and coincides with that at Terre Adélie; however, there is no evidence of a close climatic relationship between Terre Adélie and the West Antarctic Peninsula region and environmental teleconnections between the two sites have not been described.

Thirdly, as the numbers of tourists visiting Antarctica have increased, concerns have been expressed about the potential disturbance caused by visitors. Disturbance may be caused by a number of activities, including by visitors approaching too close to penguin colonies [46]. Despite the diversity of species, experimental approaches and observations, there is still little general consensus about the conclusions drawn from a range of penguin disturbance studies. As a result, there is still considerable uncertainty about the magnitude and significance of tourist impacts upon breeding penguins. However, in the case of Emperor Island, tourism can be ruled out as a possible cause of colony decline. The island has been an Antarctic Specially Protected Area since 1965 (www.ats.aq/documents/recatt/Att178_e.pdf; accessed 7 October 2010) and apart from difficulties of access during the breeding season, tourism and other visits (except for purposes of specific scientific research) are not permitted.

Fourthly, the demise of the emperor colony could have been because of disease. For example, disease was implicated in a die-off of Adélie penguins in the vicinity of Mawson Station (67° 36′ S, 62° 52′ E) during 2001 [47]. Such die-offs are thought to be rare [47] and indeed no infectious pathogens were ever isolated from the Mawson event. However, such factors cannot be discounted. Unfortunately, no information is available for Emperor Island to investigate whether this was a possible cause.

Finally, marine harvesting has been shown to have important impacts on penguins; commercial fisheries have reduced the carrying capacity of the Benguela ecosystem for penguins to only 10–20% of what it was in the 1920s [48]. However, though harvesting for Antarctic krill and finfish has been underway in the Antarctic since the late 1960s, the closest reported harvesting has been distant from Emperor Island occurring some 400 km to the north close to the South Shetland Islands and in Bransfield Strait [49], [50]. Thus, no harvesting has been recorded in the vicinity of the colony.

In summary, we believe that tourism (or other visits), and fishing can be discounted as causes leading to change at the emperor colony on Emperor Island. However, the other alternative hypotheses remain feasible and cannot be ruled out entirely, although on examining each hypothesis in turn we have no evidence that increased snowfall, extreme events, or disease played any part in the decline of this colony.

### Conclusion

We suggest that the Emperor Island colony might be a sentinel for those other colonies in the West Antarctic recently discovered [8] and even those elsewhere in the Antarctic as regional temperatures continue to rise [28] affecting changes in sea ice dynamics, formation of predictable or persistent polynyas, snowfall, wind characteristics, extreme weather events, foodwebs, and other ecological factors important for emperor penguin population processes.

The location of this colony suggests that ecological models, even simple conceptual models such as shown in our partial ecogram (Figure 6), are important for formulating predictions about future population outcomes. Such models are always site and context specific but they are critical in the absence of adequate monitoring data that would enable rigorous empirical analysis or innovative population modelling [3]. The location of the Emperor Island colony highlights that breeding habitat is only one of the factors important in such population analyses and that other factors are also essential to consider.

Our more specific conclusions include:

1. Our study is severely limited by a lack of consistent long-term monitoring information documenting, *inter alia*, penguin breeding parameters, prey species, predation and fast ice coverage. However, the available data suggest that changing sea ice conditions (plausibly both pack ice and fast ice) have had a major impact on a colony that was already vulnerable and towards, but not at, the edge of the species geographic range.
2. The principle factors leading to the decline are now difficult to disentangle and might involve factors that operate either at the breeding colony (e.g. increased snowfall, altered wind characteristics, increased predation) and/or within the seasonal foraging ambit of the population (e.g. altered foodweb characteristics).
3. Extreme weather events may have contributed to the colony decline, but records are inadequate to determine whether this was an important factor.
4. The Emperor Island colony now apparently no longer exists and any future analysis will continue to be bound by existing (and sparse) data.
5. Given their lack of agility, emperors would be limited by terrain and elevation, nevertheless, shifting their breeding habitat to low-lying accessible land is not necessarily a long term survival option for the species.
